# Electricity in Gleet and Impotence

**Published:** 1888-08

**Authors:** W. E. Steavenson


ELECTRICITY IN GLEET AND IMPOTENCE.—In the treatment of *gleet* electricity has been found very successful. It is used somewhat in the same way as medicated bougies. The metal part of a vesical electrode or electrical bougie, such as is used in the treatment of stricture, is held against the sore unhealed surface, which keeps up the discharge, and which is frequently to be found on the bladder side of a stricture. The electrode is made negative, and is moved slowly backward and forward over the sore surface for about two or three minutes, with a current strength of five milliampères. The electrode connected with the positive pole is placed on some indifferent part of the body, but by preference over the lumbar enlargement of the cord, as possibly the effect of the electricity upon the nervous supply of the urethra may be beneficial. In the treatment of gleet, no doubt, the electrolytic property of the current is the chief agent at work. The unhealthy ulcerated surface on which the gleet depends is decomposed or altered in such a way as to put it into a condition in which it will heal. *Impotence*, or inability to perform the sexual act, is also often greatly improved by electricity. It is employed for this purpose much more frequently in America and France than in our own country, but I have seen great improvement take place by "central galvanization," as described in the first paper of this series, chiefly by applying the negative electrode to the lower part of the spinal cord.—*W. E. Steavenson, M. D., M. R. C. P., in the Provincial Med. Journal.*

